# Evaluating a clinical decision support point of care instrument in low resource setting

**DOI:** 10.1186/s12911-023-02144-0

**Published:** 2023-03-30

**Authors:** Geletaw Sahle Tegenaw, Demisew Amenu, Girum Ketema, Frank Verbeke, Jan Cornelis, Bart Jansen

**Affiliations:** 1 Department of Electronics and Informatics (ETRO), Vrije Universiteit Brussel (VUB), Pleinlaan 2, 1050 Brussels, Belgium; 2 Faculty of Computing, JiT, Jimma University, Jimma, Ethiopia; 3 Department of Obstetrics and Gynecology, College of Health Science, Jimma University, Jimma, Ethiopia; 4 Imec, Kapeldreef 75, 3001 Leuven, Belgium

**Keywords:** Low resource settings, Clinical decision support system, Computer aided decision support system, Clinical pathways, Evaluation, Point of care, Low cost

## Abstract

**Background:**

Clinical pathways are one of the main tools to manage the health care’s quality and concerned with the standardization of care processes. They have been used to help frontline healthcare workers by presenting summarized evidence and generating clinical workflows involving a series of tasks performed by various people within and between work environments to deliver care. Integrating clinical pathways into Clinical Decision Support Systems (CDSSs) is a common practice today. However, in a low-resource setting (LRS), this kind of decision support systems is often not readily accessible or even not available. To fill this gap, we developed a computer aided CDSS that swiftly identifies which cases require a referral and which ones may be managed locally. The computer aided CDSS is designed primarily for use in primary care settings for maternal and childcare services, namely for pregnant patients, antenatal and postnatal care. The purpose of this paper is to assess the user acceptance of the computer aided CDSS at the point of care in LRSs.

**Methods:**

For evaluation, we used a total of 22 parameters structured in to six major categories, namely “ease of use, system quality, information quality, decision changes, process changes, and user acceptance.” Based on these parameters, the caregivers from Jimma Health Center's Maternal and Child Health Service Unit evaluated the acceptability of a computer aided CDSS. The respondents were asked to express their level of agreement using 22 parameters in a think-aloud approach. The evaluation was conducted in the caregiver's spare-time after the clinical decision. It was based on eighteen cases over the course of two days. The respondents were then asked to score their level of agreement with some statements on a five-point scale: strongly disagree, disagree, neutral, agree, and strongly agree.

**Results:**

The CDSS received a favorable agreement score in all six categories by obtaining primarily strongly agree and agree responses. In contrast, a follow-up interview revealed a variety of reasons for disagreement based on the neutral, disagree, and strongly disagree responses.

**Conclusions:**

Though the study had a positive outcome, it was limited to the Jimma Health Center Maternal and Childcare Unit, and hence a wider scale evaluation and longitudinal measurements, including computer aided CDSS usage frequency, speed of operation and impact on intervention time are needed.

**Supplementary Information:**

The online version contains supplementary material available at 10.1186/s12911-023-02144-0.

## Background

Health informatics research has produced a variety of technologies that aid the use and the production of health information. This includes automated clinical pathways (evidence-based recommendations and evidence-informed processes that integrate research evidence alongside practitioner expertise and the patient’s experience). They can reduce cost and risk at the point of care (POC) [1]. Clinical pathways (CPs) have been used to bridge the evidence/practice divide and to aid frontline healthcare workers by providing summarized evidence [2, 3]. Many clinical decision support tools, however, have remained out of reach for low-income countries (LRCs). The hurdles in care delivery for LRSs are complex, and study findings revealed that the national pyramidal health structure is "weakened at the bottom of the pyramid, and disproportionately favoring national hospitals" [4]. Furthermore, rising medical costs and scarcity of appropriate equipment, demographic challenges, inadequate infrastructure, coverage, lack of equitable health distribution, privacy and security, resource constraints, and a low literacy rate are all issues that have yet to be addressed in the implementation of an efficient integrated health information system, clinical care, and guidelines [5–7]. Moreover, our case study illustrates that existing paper-based point-of-care instruments have the "disadvantage of being non-interactive and difficult to use for retrieval of relevant clinical information, summarizing the patient history, creating a patient flow diagram, diagnosing all potential underlying diseases, and ultimately delivering optimal clinical pathways” [8]. As a result, putting evidence into practice is a very difficult matter.

To close this gap, we developed a computer aided CDSS to quickly identify cases that require referral and those that can be treated locally. To demonstrate the findings, we chose use cases of pregnant patients, routine antenatal and postnatal care, based on Ethiopian primary healthcare workflow and guidelines [9]. Within this setting the computer aided CDSS was developed using a hybrid algorithm to generate clinical pathways. The details of the design, development process, and the outcome are described in [10]. Our study is one of the rare initiatives that have opted to develop a computer aided CDSS, specifically for LRS. It focuses on primary healthcare in particular. To deal with data readiness and infrastructure, for example, it operates with limited input (clinical signs and symptoms) and progressively updates the generated clinical pathway when more information becomes available, and it was deployed on low-cost devices and accessible from a smart phone via mobile data or wireless networks. The computer-aided CDSS offers an automated, interactive, dynamic, and data-driven solution to assist front-line workers active under LRS conditions in the primary health setting.

The goal of this paper is to explain the user acceptance of the computer aided CDSS at the POC in a LRS. Moreover, we employed an artificial intelligence-enabled clinical decision support system framework (Ji, Mengting, et al. 2021 [11]) to assess the user acceptability of the computer aided CDSS.

## Methods

### Research & development options

The framework for evaluating an artificial intelligence–enabled clinical decision support system was developed based on Ji, Mengting, et al. 2021 [11]. We customized it to our needs, and a research protocol was developed. Additional file 1: Appendix I contains the protocol's details. For reporting the computer-aided CDSS evaluation, the DECIDE-AI reporting guidelines were used [12].

### Site selection

The evaluation was carried out at Jimma Health Center Maternal and Child Health Service Unit, Jimma, Ethiopia. In Ethiopia, health centers typically serviced 15,000 to 25,000 people in rural settings and up to 40.000 people in urban settings [13]. Jimma Health Center is not an exception. The health center acts as a focal point by handling both inpatient and outpatient cases. It accepts referral cases from community health posts as well as it refers cases and assigns patients to the primary hospitals (Jimma University Specialized Hospital and Shanan Gibe General Hospital).

To manage frequently collected public health facility data in Ethiopia, the electronic Community Health Information System (eCHIS) and District Health Information Software (DHIS2) were introduced [14–17]. These tools are mainly used for collecting and reporting public health facility data. However, decision-support systems, electronic health records, the infrastructure for exchanging health information, and other similar technologies were not readily available to or used by frontline workers. During the need analysis [8, 18], we studied the clinical guidelines, the patient card-sheet, and referral-out registration logbook at Jimma Health Center. Paper-based clinical guidelines, card sheets, and referral registration log sheets are the only readily available resources for assisting frontline workers and their decisions [8, 18]. However, none of them are automated, interactive or dynamic. To capture the required information from the existing paper-based instrument takes much time and only contain limited information. Overall, it’s challenging to “capture and summarize the required clinical data, process it in a consistent manner, construct a patient flow sheet to monitor and record the progress of care” [8, 18]. It was difficult to audit records and track changes because of the inconsistent handwriting and layout. Furthermore, the health information transformation plan states that there are no known or accessible decision-support tools that generate and promote evidence-based decisions, including a lack of decision support tools that incorporate program and clinical guidelines, a lack of automated condition-specific order sets and documentation to facilitate decisions, and a lack of knowledge management systems [19, 20].

### Participants

Caregivers who work at Jimma Health Center in the maternal and child healthcare unit or department were eligible to participate in the evaluation experiment. The caregivers volunteered to participate and gave their consent after receiving a description of the study and the computer aided CDSS. A flexible organization of the evaluation was needed to attract enough participants. The evaluation was done during the participants’ spare-time because the number of health professionals at the health center's maternal and child healthcare unit is limited, and they were fully occupied by completing their ordinary daily activities so that it was not feasible to include the evaluation in their daily practice. As a result, the evaluation procedure for the computer aided CDSS was carried out after the clinical decision was made rather than during the real-time decision-making process. Furthermore, instead of an instant patient-by-patient evaluation, the evaluation was completed a posteriori over the course of a half-day, and the computer aided CDSS had no impact on the care given. The participants were given a guide with detailed step-by-step instructions on how to use the computer aided CDSS to assist them in better preparing for the activity after clarifying the goal and obtaining consent.

Furthermore, the following factors were considered when determining the number of participants in the computer aided CDSS evaluation study:

- I. Ethiopia has a health workforce that is far below the minimum standard [21, 22]. The number of health professionals at the health center level is insufficient. The number of maternal and child healthcare unit caregivers at the health center is very limited, five to seven care givers on average and exceptionally some more.
- II. Consulting the literature, and given the small number of care givers, we adopted the "rule of thumb 4 ± 1" suggested when there are financial restrictions and a small number of participants. The magic number of five (4 ± 1) evaluators effectively recognizes the majority of usability issues as reported in [23, 24] for an evaluation in well constrained context. Three to five evaluators, for example, identify 85 to 91% of usability issues [23, 24]. Therefore, we made the assumption in our work that our experts can identify the vast majority of usability problems. Our computer aided CDSS evaluation studies were conducted with experts in primary care settings for maternal and childcare services, namely for pregnant patients, antenatal and postnatal care. Overall, we considered all caregivers who volunteered to take part in the computer aided CDSS evaluation at Jimma Health Center's maternal and child healthcare unit.

### Computer aided clinical decision support POC

**Fig. 1:**
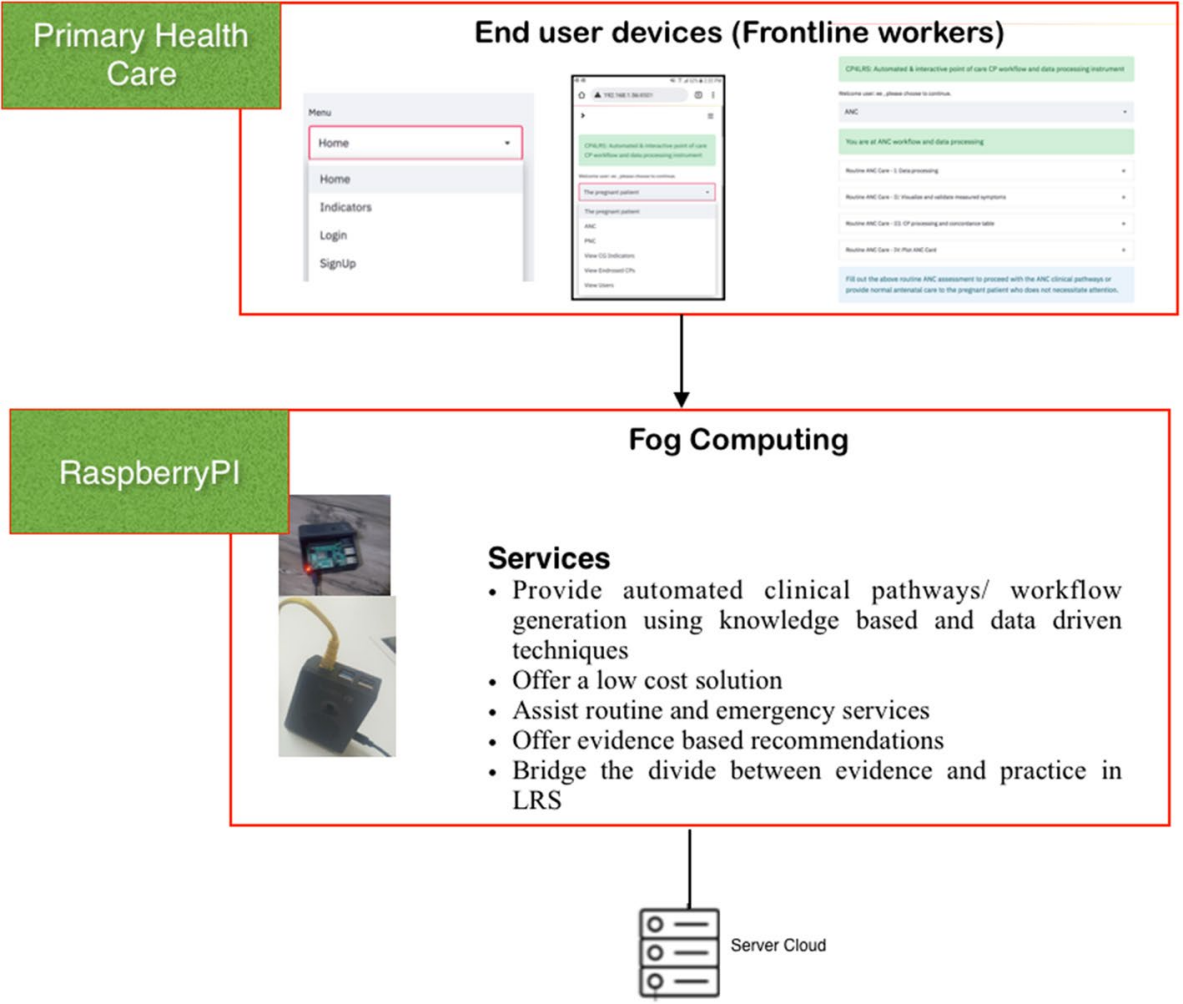
Low cost architecture

**Fig. 2:**
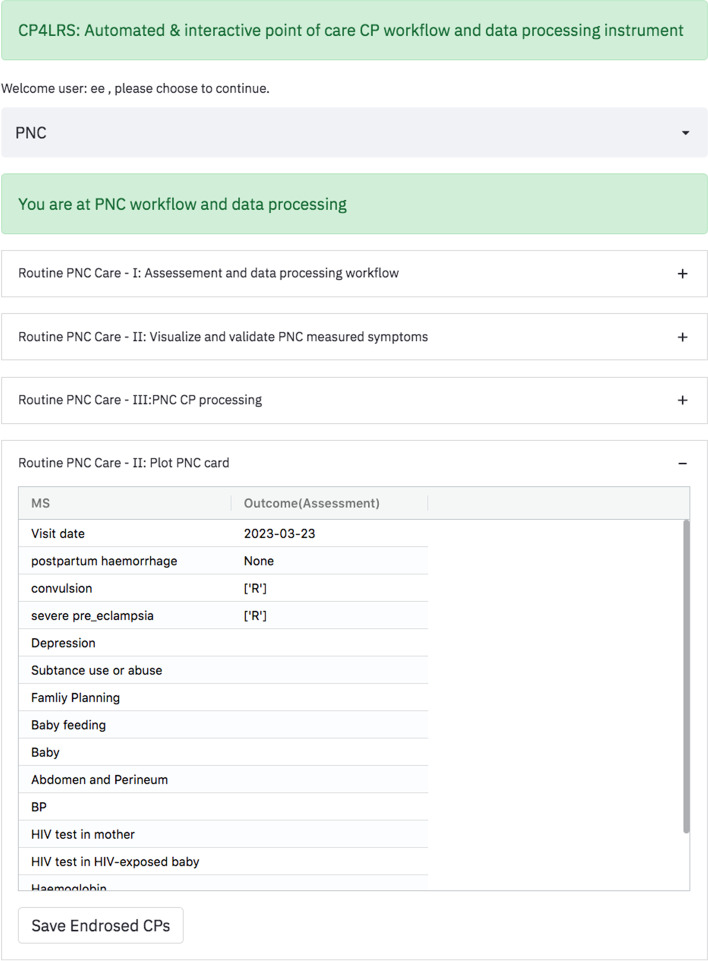
Sample screenshot for postnatal clinical workflow

The computer aided CDSS was developed to promote high-quality care and assist healthcare workers in identifying referral and locally treatable cases. Integrating the knowledge-based approaches with the data-driven techniques is the core principle behind computer aided CDSS development [10]. This delivers the flexibility to dynamically map and evaluate knowledge in the local context. It also supports the use of historical evidence to adjust or re-adjust the order in the priorities of the knowledge-based CPs' decisions using the concordance table (a multi-criteria decision analysis). Bayesian probabilistic learning was combined with automated and dynamic knowledge-based approaches on the Jimma Health Center "pregnancy, childbearing, and family planning" dataset, providing a satisfying result [10]. Then, the computer aided CDSS was deployed in a low-cost fog computing architecture [25–27]. Raspberry Pi 4 Model B, which has a quad-core 64-bit processor and 4 GB of RAM was used as a platform. The computer aided CDSS data entry and processing was designed in a wizard style in accordance with the clinical guidelines [9]. A multi-criteria decision analysis and concordance table were generated based on the measured symptoms. The overall process consists of four steps: (I) Entering measured symptoms, (II) Validating and checking the measured symptoms, (III) Processing of clinical pathways to identify referral and locally treated cases, (IV) Selecting and endorsing the clinical pathways that have been generated, and (V) Saving the endorsed CP for future reference. Furthermore, automated antenatal (or postnatal) card plotting was done after the selection or endorsement of CPs. The computer aided CDSS can be accessed from a smart phone, tablet PC, or laptop running a web browser via a mobile data or wireless network. The architecture of computer-aided CDSS and the sample screenshot for postnatal (PNC) clinical workflow and the data processing are shown in Figs. 1 and 2, respectively.

### Implementation

To evaluate the computer aided CDSS, we adopted 22 parameters from the evaluation framework of Ji, Mengting, et al. 2021 [11]. We only considered 22 of the 28 parameters because outcome changes (i.e. Change in clinical outcomes and Change in patient-reported outcomes), service quality (i.e. operation and maintenance, and information updating to keep timeliness), and process change productivity (i.e. productivity) were outside the scope of our study, and the computer aided CDSS was evaluated after the clinical decision was made. In addition, the variables Satisfaction of system quality, Satisfaction of information quality, and Satisfaction of service quality were difficult to distinguish, and hence we aggregated them as “Overall Satisfaction”.

The think-aloud protocol was followed while the participant uses the computer aided CDSS. The system was evaluated after the clinical decision was made using the concurrent think-aloud approach. In a thinking aloud test (TA-Test), participants were asked to use the computer aided CDSS while continuously thinking out loud [28]. Prior to the evaluation, a presentation describing the purpose of the evaluation and a computer-aided CDSS demonstration were delivered. During the think aloud-based and computer aided CDSS evaluation, the participant was not audio recorded. However, if participants felt uncertain and uncomfortable, they would "think aloud," and the researcher would document their thoughts.

Next, the caregivers at Jimma Health Center Maternal and Child Health Service Unit completed the questionnaire, which is organized around a kind of psychometric response scale [29] in which respondents express their level of agreement to a statement in five scores: (1) strongly disagree, (2) disagree, (3) neutral, (4) agree, and (5) strongly agree. In addition, when the evaluator responded, "strongly disagree, disagree, or neutral," we did a follow up by asking for more details on the reasons of their low scores in an interview. The interview was recorded and later reviewed for further computer aided CDSS improvement. The audio recordings of the follow-up interview were transcribed into verbatim text. The follow-up interview was conducted in Amharic and was then translated into English.

The questionnaire was structured into five sections with a total of 22 questions to validate and measure the perceptions on the instrument's characteristics in the following order: ease of use (6/22), system quality (2/22), information quality (2/22), decision changes (2/22), process changes (5/22), and user acceptance (5/22) [11]. The variables "learnability, operability, user interface, data entry, advice to display, and legibility" were used to assess ease of use. System quality relates to the performance of the computer aided CDSS system and the needed functionality as measured by "Response time and Stability”. Information quality denotes the computer aided CDSS’s capacity to conduct actions with suitable evidence within acceptable time frames as well as data protection, expressed in two factors, namely "Security and CP performance”. Then, decision changes were evaluated based on variables "Change in order behavior and Change in CP" to evaluate the computer aided CDSS’s capabilities to allow for real-time interactions, as well as the computer aided CDSS’s relevance. The variables "Effectiveness, Overall usefulness, Adherence to standards, Medical Quality, and User knowledge and skills" were then used to evaluate "Process changes”. Finally, user acceptance was assessed using the variables "usage, expectation confirmation, satisfaction over quality, overall stratification, and intention to use”. A more detailed description of the 22 evaluation parameters is given in Additional file 1: Appendix I. To compute the respondent response in each of the 6 sections, an averaged agreement score was calculated. The agreement score was computed using the responses "Agree" and "Strongly agree”. The disagreement score was computed on the responses “Strongly disagree”, “Agree” and “Neutral” (see Table 1).

**Table 1:** Summary of agreement scores based on respondent response agreement

Categories	Parameters	Agreement score (Agree + Strongly agree)	Disagreement score (Strongly disagree + disagree + neutral)	Total agreement score
Ease of Use	Learnability	0.75 (3/4)	0.25	4.25/6
	Operability	0.75 (3/4)	0.25	
	User Interface	0.75 (3/4)	0.25	
	Data Entry	0.75 (3/4)	0.25	
	Advice to display	0.75 (3/4)	0.25	
	Legibility	0.5 (2/4)	0.5 (2/4)	
System Quality	Response time	0.75 (3/4)	0.25 (1/4)	1.25/2
	Stability	0.5 (2/4)	0.5 (2/4)	
Information Quality	Security	0.75 (3/4)	0.25 (1/4)	1.5/2
	CP Performance	0.75 (3/4)	0.25 (1/4)	
Decision Change	Change in order behavior	0.5 (2/4)	0.5 (2/4)	1.5/2
	Change in CP	1 (4/4)	0	
Process Change	Effectiveness	0.75 (3/4)	0.25 (1/4)	4/5
	Overall Usefulness	0.75 (3/4)	0.25 (1/4)	
	Adherence to standards	1 (4/4)	0	
	Medical quality	0.75 (3/4)	0.25 (1/4)	
	User knowledge and skills	0.75 (3/4)	0.25 (1/4)	
User Acceptance	Usage	0.75 (3/4)	0.25 (1/4)	3.75/5
	Expectation confirmations	0.50 (2/4)	0.50 (2/4)	
	Satisfaction of overall quality	0.75 (3/4)	0.25 (1/4)	
	Overall satisfaction	0.75 (3/4)	0.25 (1/4)	
	Intention to use	1 (4/4)	0	
Total		16.25	5.75	

Furthermore, the questionnaire was translated into Amharic. A freelance and experienced translator then reviewed the translated questionnaire to resolve any discrepancies between the original English version and the translated Amharic questionnaire. The questionnaire was accessible for submission through mobile phone, laptop, or paper-based format. We prefer mobile or laptop-based formats to paper-based formats, the latter being used exceptionally. The English version of the questionnaire is included in Additional file 1: Appendix I. In addition, the automated version of the questionnaire is available on Github.^1^ Python and Streamlit framework were used to automate the questionnaire. Following submission, the automated questionnaire filters responses such as "strongly disagree," "disagree," and "neutral" for a follow-up interview.

### Outcome

The primary outcome of the study was the evaluation of the user acceptance of the developed computer aided CDSS in a LRS. Computer-aided CDSS’s ease of use, system quality, information quality, decision changes, process changes, and user acceptance were explicitly addressed.

### Safety, errors, and human factors

Since the evaluation was conducted after the clinical decision was made, there was no risk for the patient safety. Furthermore, an artificial intelligence-enabled clinical decision support system framework [11] was used, and the assessment was carried out using this framework, with participation from the caregiver at the health center.

### Analysis

The study aimed at evaluating the user acceptance of a computer aided CDSS in a LRS and the findings were analyzed to gain insight and uncover common patterns to identify future actions. First, we analyzed the time taken to fill-in the questionnaire, to cross-check the plausibility and credibility of the evaluation. Then, the analysis was conducted based on ease of use, system quality, information quality, decision changes, process changes, and user acceptance of the computer aided CDSS. Moreover, we used a Python tool and Microsoft Excel for data processing and analysis.

This study reported the verbatim text and participant comments in two ways: (I). For verbatim quotations from a single participant, direct quotation marks were used, and (II). If more than one participant made the same comment on a specific topic the researcher paraphrased and summarized it without making use of quotes.

### Ethics

Initially, we got ethical permission from Jimma University Institute of Health's Institutional Review Board. The data was then collected and processed anonymously following the Jimma health center signed consent during need analysis and computer aided CDSS development. The clinical guideline was employed as a gold standard for validating the automated and data-driven generated clinical pathways, ensuring the fairness of the results. Then, to assure the authenticity and integrity of the computer aided CDSS evaluation, we employed an artificial intelligence evaluation framework for computer-aided CDSS evaluation [11] and DECIDE-AI reporting guidelines [12] for reporting CDSS evaluation results. Finally, the computer-aided CDSS evaluation was conducted after the clinical decision was made to ensure that the computer-aided CDSS had no impact on the real decision-making process. Moreover, personal information exclusively used for questionnaire verification did not appear in the reporting and in the results. In general, we are committed to protecting personal information and respecting privacy as per the agreement consent.

### Patient involvement

Since the primary purpose of the computer aided CDSS is to help caregivers and frontline workers identify referral and treatable cases at the health center, the patient was not directly involved in the user-acceptance evaluation study. In addition, the option has been taken to conduct the evaluation after the clinical decision for the patient had been taken.

## Results

Caregivers from Jimma Health Center's Maternal and Child Health Service Unit evaluated the acceptability of a developed computer aided CDSS at the POC for two days. The evaluation was carried out at Jimma Health Center between June 4 and 6, 2022. The caregivers assessed the computer aided CDSS by responding to a total of 22 questions divided into six categories, namely ease of use, system quality, information quality, decision changes, process changes, and user acceptance.

The maternal and child healthcare unit caregivers at the health center were limited to five active caregivers during the computer aided CDSS evaluation. Four of these caregivers participated in the computer aided CDSS evaluation. One of the caregivers was unavailable during the evaluation. All of the respondents were female who have worked for the maternal and childcare health center unit and department.

There were eighteen cases in the Maternal and Childcare Unit (during the first day: ten cases, the second day: eight cases). The longest duration to complete answering the questions in the questionnaire was 98 min and the shortest was 31 min, with an average time of 57.75 min. Though the participants use their spare time for evaluation, we observed that they spent more time in the afternoon (or night) session than they did in the morning (or mid-day).

Based on the responses, the computer aided CDSS received an average user acceptability score of 3.75 out of 5; an ease-of-use score of 4.25 out of 6; a process change score of 4 out of 5; and a system quality score of 1.25 out of 2. Information quality and decision change received a score of 1.5 out of 2. Table 1 shows the complete results, and a summary of the respondent agreement scores.

**Fig. 3:**
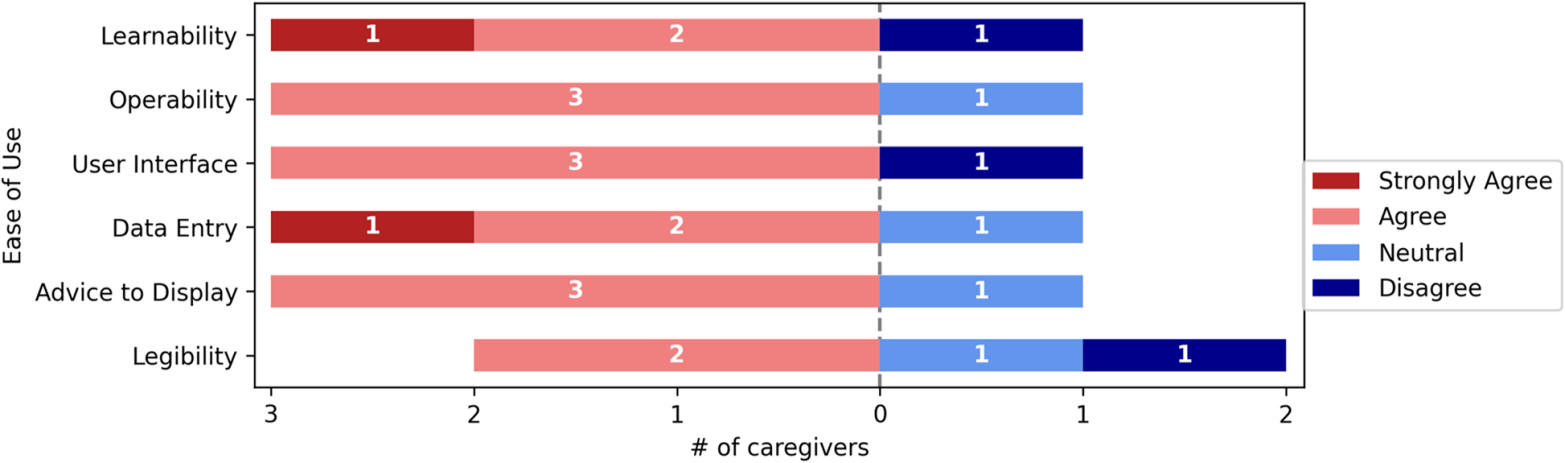
Ease of use

**Fig. 4:**
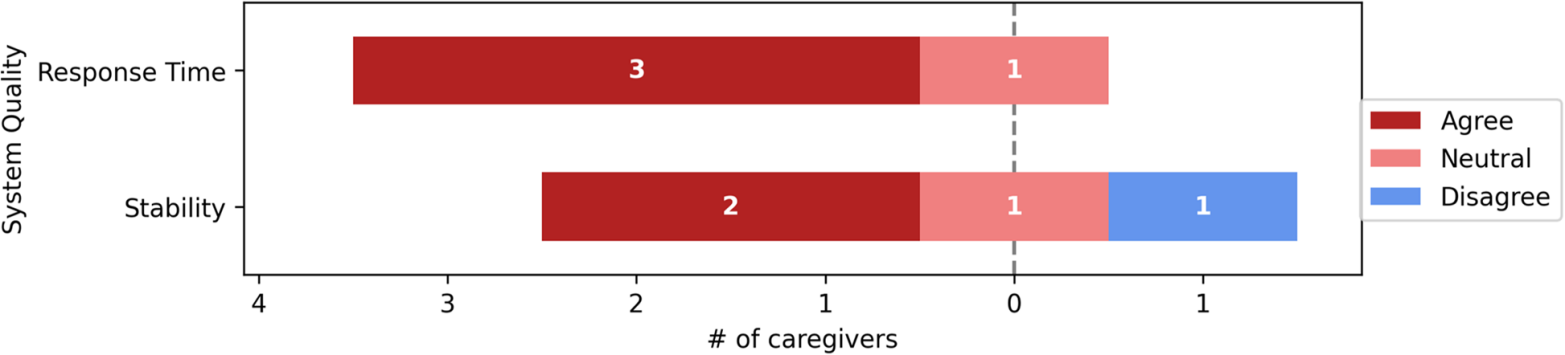
System quality

**Fig. 5:**
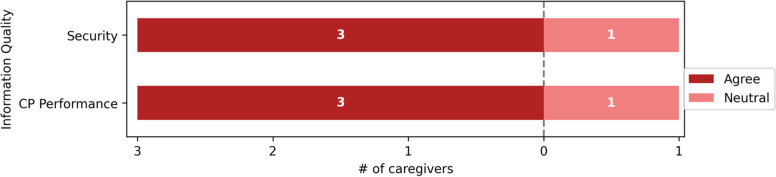
Information quality

**Fig. 6:**
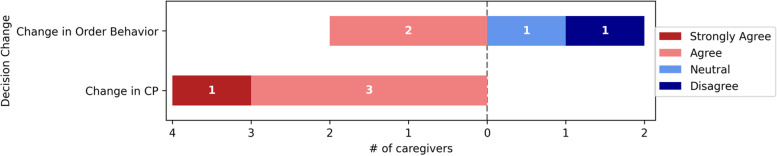
Decision change

**Fig. 7:**
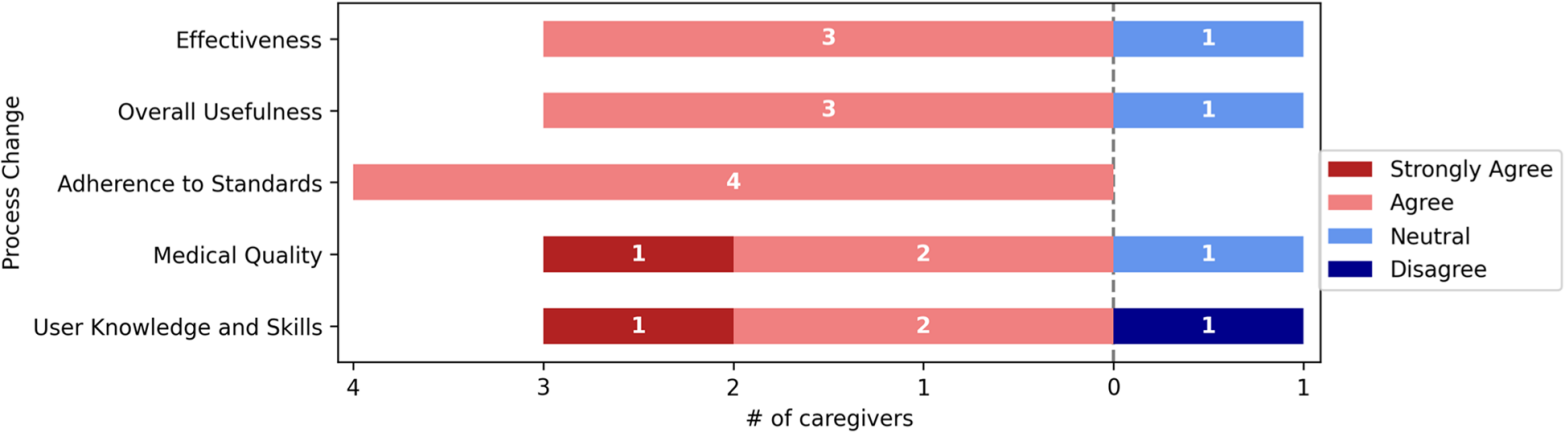
Process change

**Fig. 8:**
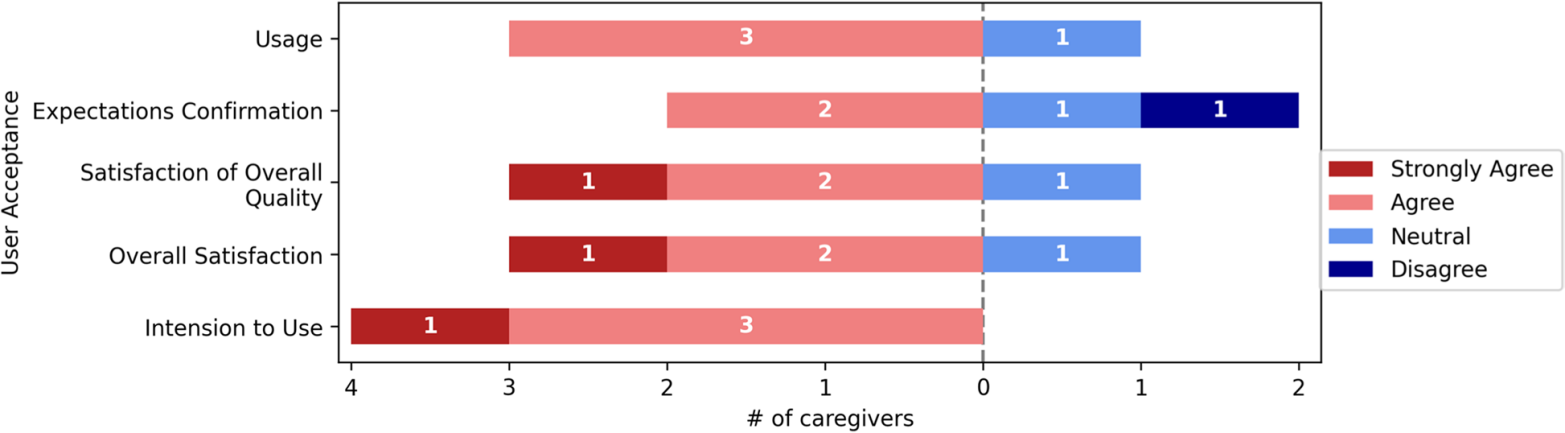
User acceptance

Overall, nurses were comfortable using the computer aided CDSS. Figures 3, 4, 5, 6, 7 and 8 depict the detailed result of the computer aided CDSS evaluation. Computer aided CDSS users' responses were categorized as Ease of use, System quality, Information quality, Decision change, Process Change, and User Acceptance.

### Ease of use

Only one respondent strongly agreed with the computer aided CDSS Learnability and Data Entry. 3/4 (75%) of respondents agreed on the operability, user Interface, and advice to display. Learnability, Data Entry, and Legibility obtained a 2/4(50%) agreement score from respondents. Figure 3 depicts the respondents’ scoring results for the category ease of use.

Generally, the participants were given 4.25 out of 6 for Ease of Use of the computer aided CDSS. Respondents, said the following:

- The first respondent stated, "I have two cases with Blood Pressure (BP) readings of 100/70 and 110/70, however, the two values were not available in the drop-down option for selecting values.”
- We feel that further instruction and guidance are essential for me to feel completely compliant with the computer-aided CDSS [Respondents 2 and 3].
- Respondent 4 stated that:”The entire output information in the table did not show until I made them full screen.”

### System quality

3/4(75%) and 2/4(50%) of the respondents were satisfied with the computer aided CDSS response time and stability, respectively. In contrast, one respondent was neutral about the response time and stability. Furthermore, one respondent disagreed with the computer aided CDS stability. Figure 4 depicts the System Quality respondents' outcome.

When using the computer-aided CDSS, participants expressed the importance of System Quality.

- Since the computer aided CDSS lacked user-type choices, we were concerned about its quality. [Respondents 1 and 2].
- "I wish it had an offline version, when the internet connection was lost or disrupted, the computer-aided CDSS was unresponsive”. [Respondent 4

### Information quality

On the Information Quality, 3/4(75%) of the respondents were satisfied with security and CP Performance. In contrast, one respondent was neutral. Figure 5 illustrates further details about the Information Quality responses.

### Decision change

All respondents were satisfied with the Change in CP, and 2/4(50%) were satisfied with the Change in order behavior. Figure 6 presents further details about the Decision Change responses.

When participant talking about the capability of computer aided CDSS allowing for realtime based interaction between the user and the computer-aided CDSS as well as the evidence got from the CDSS diversity and importance, respondent 2 and 4 stated that,

- Our interaction with the computer-assisted CDSS seems to be restricted, and we were unable to enter cases outside of the drop-down options. [Respondents 2 and 4]

### Process change

The evidence generated by the computer aided CDSS’s adherence to standards, such as the Ethiopian primary healthcare guidelines, was supported by the respondents. 3/4(75%) of the respondents were satisfied with the effectiveness and overall usefulness. However, one respondent were neutral in terms of effectiveness, overall usefulness, and medical quality, while one disagreed with the outcome's consistency with existing user knowledge and skills. Figure 7 shows further information about the Process Change responses.

There were participants who were concerned about the computer aided CDSS process changes.

- Based on the input, the computer-aided CDSS successfully generated results. However, we expect other sources of evidence in addition to the guidelines. We wish there were user-typed alternatives and a more flexible data entry option. [Respondents 2, 3 and 4].

### User acceptance

The overall quality, overall satisfaction, and intention to use the computer aided CDSS, obtained a strongly agreed score from 1/4(25%) of the respondents; 3/4(75%) of the respondents agreed on the usage and intention to use of the computer aided CDSS. Usage, expectation confirmation, satisfaction with overall quality, and overall satisfaction were all neutral for 1/4(25%) of the respondents. Furthermore, one respondent disagreed with the expectation confirmation of the computer aided CDSS. Figure 8 shows further information about the User Acceptance responses.

Participants expressed an interest in using computer-assisted CDSS in their daily routine. Participants were positive about the "use, expectation confirmations, satisfaction with overall quality, and overall satisfaction" of the computer-aided CDSS. On the other hand,

- Respondents 2 and 3 stated that,
  we have minimal prior experience, but in order to completely accept the computer-aided CDSS, we need flexible data entry as well as more sources of evidence.
- Respondent 4 also stated that,
  “I expect some type of advice to show and offline support version to completely approve the computer CDSS”.

Overall, the participants were positive on computer-aided CDSS “Intention to use, change in CP, and adherence to the standards”.

- "Despite the limitations noted above, I believe it will be addressed in the next version” [Respondent 1].
- We found the computer-aided CDSS useful and will use it again if we have access since it corresponds to the standards and clinical guidelines, and the results were apparent to us. [Respondents 1, 2, 3 and 4].

In summary, the computer aided CDSS had a positive agreement score. User acceptance achieved a 3.75 out of 5 agreement score. However, caregivers appear to be concerned, as evidenced by a disagreement score of 1.75 out of 6 on the computer aided CDSS's Ease of Use; 0.75 out of 2 on System Quality; 0.5 out of 2 on Information Quality; 0.5 out of 2 on Decision Change; 1 out of 4 on Process Change; and 1.25 out of 5 on User Acceptance. A variety of disagreements has been revealed during a follow up interview across each of the categories. With the exception of Change in CP, Adherence to standards, and Intention to use, most computer aided CDSS evaluation parameters received a disagreement score of 1/4(25%) to 2/4(50%) from respondents. Table 2 summarizes the parameters and the respondents' reasons for disagreement. The letters R1, R2, R3, and R4 in Table 2 denote Respondent 1, Respondent 2, Respondent 3, and Respondent 4. R2, for example, disagreed with the computer aided CDSS’s Perceived Ease of Use evaluation metrics based on learnability parameters.

**Table 2:** Summary of respondents’ disagreement reasons by parameters

Categories	Respondents' disagreement			
	RI	R2	R3	R4
Perceived Ease of Use		Learnability		
			Operability	
	User Interface			
		Data Entry		
				Advice to display
		Legibility		
System Quality				Response time
	Stability	Stability		
Information Quality		Security		
				CP Performance
Decision Changes		Change in order behavior	Change in order behavior	
Process Changes		Effectiveness		
			Overall usefulness	
	Medical quality			
		User knowledge and skills		
Acceptance	Usage			
		Expectations confirmation	Expectations confirmation	
		Satisfaction of quality		
				Overall satisfaction

In all, Table 2 summarizes the respondents' reports of disagreement. The details of the disagreement reason, on the other hand, were transcribed directly from the follow-up interview recording. Table 3 contains information on the reasons for disagreement, which are generally transcribed verbatim from the interview and were reported as extracted and summarized reasons of disagreement from the follow-up interview.

**Table 3:** CDSS computer-aided disagreement reasons retrieved from the follow-up interview

Categories	Parameters	Respondents				Reason for disagreement: Extracted and summarized from the follow-up interview
		R1	R2	R3	R4	
Perceived Ease of Use	1. Learnability		✓			R2 observes that extra training and guidance are necessary
	2. Operability			✓		R3 comments that some help and guidance are necessary to reduce the amount of effort and time required. On the first day, dealing with the computer aided CDSS was very challenging
	3. User Interface	✓				R1 regrets that computer-assisted CDSS is confined to automated wizard creation and combo-box-based selections, and that it lacks flexible real-time interactions between the user and the computer-assisted CDSS recommendations
	4. Data Entry		✓			R2 observes and explicitly emphasizes that the computer aided CDSS lacks flexible data entry and user typing alternatives
	5. Advice to display				✓	R4 receives table-format output, but reports that the full information and parameters are not displayed until the output window is expanded to full screen
	6. Legibility		✓	✓		Both R2 and R3 said that non-professionals would need more guidance and training to properly comprehend the system
System Quality	7. Response time				✓	R4 experienced mobile network instability during the evaluation and stated that he/she is unable to comment on response time
	8. Stability	✓	✓			Both R1 and R2 realized that the computer aided CDSS did not handle exceptions and that all inputs were strictly based on guidelines
Information Quality	9. Security		✓			R2 said explicitly that he/she is unable to comment on security since the opinion of the right experts is required
	10. CP Performance				✓	R4 feels that further study is required before concluding that the computer aided CDSS is superior to existing paper-based evidence- resources and he/she is unable to make remarks on security
Decision Changes	11. Change in order behavior		✓	✓		Both R2 and R3 said that the computer aided CDSS is restricted to automated wizard creation and combo-box based selections and lacks flexible real-time interactions between the user and the computer aided CDSS recommendations. This is the cause for their disagreement Scoring
	12. Change in CP					There are no disagreement remarks for "Change in CP"
Process Changes	13. Effectiveness		✓			Though R2 found the computer-aided CDSS process workflow and display output for completing tasks effectively as expected, R2 expects the inclusion of additional sources of evidence and output
	14. Overall usefulness			✓		The measured symptoms wizard, according to R3, is exclusively based on clinical guidelines. To explore exceptions such as input exceptions, R3 emphasized that the computer aided CDSS should include evidence other than clinical guidelines
	15. Adherence to standards					There are no disagreement remarks for “Adherence to standards"
	16. Medical quality	✓				In addition to the computer aided CDSS output workflow and evidence, R1 suggests including additional evidence beyond clinical guidelines
	17. User knowledge and skills		✓			According to R2, the user's ability to explore exceptions in locally treatable cases is good. The computer aided CDSS, on the other hand, limits flexibility in data entry and requires more historical and diverse evidence from the patient card sheet
Acceptance	18. Usage	✓				R1 declares neutrality due to her/his dissatisfaction with the user interface, stability, and medical quality
	19. Expectations confirmation		✓	✓		On computer aided CDSS expectations confirmation, both R2 and R3 were not satisfied. Both claim to have had no prior intensive computer aided CDSS experience other than minimal exposure to the District Health Information Software (DHIS) and electronic community health information system (eCHIS) software. However, R2 was not fully satisfied on the computer aided CDSS in terms of learnability, data entry, legibility, stability, change in order behavior, and effectiveness. R3 were dissatisfied with Operability, Change in order behavior, and Overall usefulness
	20. Satisfaction of overall quality		✓			The computer aided CDSS dissatisfaction with "learnability, data entry, legibility, stability, change in order behavior, and effectiveness" resulted in R2 neutrality on "satisfaction of overall quality”
	21. Overall satisfaction				✓	The R3 neutrality on computer aided CDSS originated as a result of dissatisfaction with "Advice to display, Response time, and CP Performance."
	22. Intension to use					There are no disagreement remarks for “Intension to use"

## Discussion

### Principal findings

The computer aided CDSS received a positive overall review, based on the average scores in all six categories. Even though this study attempted to evaluate the clinical decision support point of care instrument in a low-resource setting and obtained a favorable agreement score in terms of "Ease of use, System quality, Information quality, Decision change, Process Change, and User Acceptance," some respondents disagreed on this. (See Table 3).

#### Ease of use

While 3/4(75%) of the respondents agreed or strongly agreed with the computer aided CDSS’s ease of use factors ("learnability, user interface, operability, data entry, and advice to display,"), 1/4(25%) disagreed.

During the follow-up interview, we identified the 1/4(25%) disagreements in each category. The 1/4(25%) disagreements on learnability were due to the fact that: although the system is straightforward and easy to use, “it still requires some help as well as guidance, the respondents are sometimes puzzled, particularly on the first day”. When commenting on the computer aided CDSS’s "operability", respondents continued to favor a neutral score on the amount of work and time necessary for the usage of the computer aided CDSS and the accomplishment of the tasks correctly. The computer aided CDSS’s User Interface is not able to accept user-typed input options other than those proposed by the system concerning measured symptoms, and all of the output parameters were not visible while viewing the concordance table on a mobile device until the concordance table was made full screen. The computer aided CDSS data entry lacks data input options for cases treated at a health facility. For example, during the day one morning evaluation, there were two cases with Blood Pressure (BP) values of 100/70 and 110/70, but those values were not available in the drop-down option for selecting values. Thus, to enable flexibility in the event of an unexpected scenario, it would be better to provide user typing alternatives. Although the computer aided CDSS favors automated wizards and recommendations, the user also expects to be able to make his/her own decisions, including overruling the computer aided CDSS recommendations that are sometimes considered unsuitable. Respondents emphasized the necessity of displaying advice and documentation based on local languages and setting options. The disagreement in legibility is 25% higher than in the other categories concerning ease of use. According to the respondents, since the computer aided CDSS is based on an automated wizard, non-AI professionals would want some guidance and training to properly understand the system.

#### System quality

The computer aided CDSS’s System Quality disagreement was composed of 1/4(25%) of the scores being neutral about the computer aided CDSS response time and stability while 25% disagreed with the computer aided CDSS stability. "There were no exceptions possible in the data entry, which hampers the flexibility because inputs were strictly based on clinical guidelines”, which results in 1/4(25%) disagreement and 1/4(25%) neutral in a follow-up interview on the stability of the computer aided CDSS. Furthermore, one respondent had mobile network instability during the computer aided CDSS evaluation submission, resulting in a 25% disagreement on response time.

#### Information quality

3/4(75%) of the respondents were satisfied with the information quality, specifically security and CP performance, while 1/4(25%) were neutral on this aspect. The disagreement resulted from security and CP performance. Even though there was a password-protected login, the respondents preferred to give neutral scores and they were unable to make remarks on security since this requires the right experts’ opinion. The respondents that gave disagreement scores believe that further research is needed to conclude that the computer aided CDSS is better than the existing evidence-based resources such as paper-based clinical guidelines and card-sheets.

#### Decision change

Though all respondents were satisfied with the Change in CP, half of them disagreed with the Change in order behavior. According to the findings of the follow-up interview, the respondents feel that the computer aided CDSS lacks flexible real-time interaction between the user and the computer aided CDSS recommendations and is too much restricted to automated wizard generation and combo-box based choices.

#### Process change

Except for standards adherence, neither of the categories satisfies 1/4(25%) of the respondents concerning the Process Change, in particular, effectiveness, overall utility, medical quality, and user knowledge and abilities. Though the computer aided CDSS provides the required output workflow based on clinical guidelines, it needs to incorporate additional evidence besides the clinical guidelines, because the user's skill in exploring exceptions in the case of locally treatable cases is good. For example, it lacks flexible data entry and requires more historical evidence from the patient card sheet, even though the majority of the existing patient card-sheets lacks documentation of comprehensive patient information.

#### User acceptance

In general, respondents were satisfied with user acceptability. Respondents were particularly interested in using the system for daily regular duties and having access to the computer aided CDSS. However, 1/4(25%) of the respondents disagreed with the computer aided CDSS user acceptability, specifically for the Usage, Satisfaction of overall quality, and Overall satisfaction parameters. The major point of disagreement was that the computer aided CDSS uses clinical guidelines as a standard, is oriented towards referral cases by nature, and so lacks data input options (and/or flexibility) for patients treated at the health center. Additionally, 50% of the respondents disagreed on Expectation confirmation parameters. The computer aided CDSS’s "Expectation confirmation" disagreement arose as the result of: (I) Respondent I: “Since I don’t have prior experience, I prefer neutral”, and (II) Respondent II: “Because I lack past experience, I prefer to disagree rather than agree or being neutral”.

### Follow-up interview summary

Table 3 presents the summary of disagreement reasons extracted during the follow up interview. The symbol "✓” indicates that there is disagreement on the specific parameters. (See Additional file 1: Appendices II for details on each parameter's questionnaire).

In conclusion, the evaluation findings including Decision Changes and Process Changes, revealed a variety of needs for the design of the next computer-aided CDSS iteration, including: (I). To improve data entry quality and manage exceptions, a user type option needs to be added to the drop-down options to provide a more flexible data entry system (II). An offline version of the computer-aided CDSS needs to be designed to promote maximum real-time interaction between the user and the computer-aided CDSS, (III). Recent literature evidence needs to be included as a source of evidence in addition to the clinical guideline and card sheet to enhance the robustness of the evidence.

### Overall strengths and limitations

This study developed a low-cost, automated, and symptom-based clinical workflow for low-resource settings and reported a promising agreement score, during the evaluation. The novel aspect of our proposed computer-aided CDSS was the inclusion of a CP algorithm that dynamically maps and validates the knowledge-based CP using data-driven approaches, primarily using Bayesian learning and incremental learning to adjust and re-adjust the decision priority using multiple criteria decision analysis [10], and implementing the algorithm in low-cost alternatives such as the Raspberry Pi 4 Model B that are suitable for low-resource settings. Furthermore, the study tried to evaluate CP outside of the hospital, a data-intensive and chronic healthcare setting, and to design and evaluate it in a primary healthcare context in low-resource settings. The computer aided CDSS also provides interactive data visualization and a clinical wizard for easy reference, appropriate clinical management, and data processing by identifying referral and locally treated cases. Despite some participants’ disagreements, the computer aided CDSS received a score of 50% to 100% agreement in all evaluation parameters. The follow-up interview also revealed substantial remarks and improvement considerations for upgrading the computer aided CDSS based on the six categories of "Ease of use, System quality, Information quality, Decision Change, Process Change, and User Acceptance”. Furthermore, the key strength of this study is its methodology. An artificial intelligence-enabled clinical decision support system was adopted for evaluation [11], and the DECIDE-AI framework was used for reporting [12], which will minimize response and reporting bias. We also track the beginning and end of the questionnaire filling process to see how long it takes, and then, upon submission, the automated evaluation framework questionnaire filters the "Strongly disagree," "disagree, and neutral" for the discussions in interviews.

There are limitations to this study. First, the evaluation was performed after the clinical decisions over the course of a half-day. Hence, the real-time decision process was not considered. Second, since the participants were limited to the Jimma Health Center maternal and childcare unit, important considerations should be made before generalizing these findings to other contexts outside of the study site. Third, the number of participants was limited, and the evaluation was restricted to one study site, which may cause a limited diversity in perspectives. While self-reported computer aided CDSS evaluation can be used, it implies a risk on introducing a bias. Some more longitudinal measurements are needed, including computer aided CDSS usage frequency, duration, and other important evaluation metrics. Finally, while this study used a standard AI-enabled evaluation framework with a thinking aloud approach to evaluate the computer aided CDSS, the computer aided CDSS long-term use and impact could not be evaluated. Furthermore, it’s important to consider also other suitable “discount usability methods” for evaluating the user acceptance of computer aided CDSS in low resource settings.

## Conclusion

The user acceptance evaluation of a computer aided CDSS at the point of care in LRSs was carried out using an artificial intelligence-enabled clinical decision support system framework. Respondents were asked to express their level of agreement using 22 parameters in a think-aloud approach. The evaluation criteria were categorized into six categories: ease of use, system quality, information quality, decision changes, process changes and user acceptance (see Additional file 1: Appendix 1). Despite considerable disagreement among participants, the computer aided CDSS achieved higher-than-average scores in all six categories, namely user acceptability (3.75 out of 5), ease-of-use (4.25 out of 6), process change (4 out of 5), and system quality (1.25 out of 2). The score for information quality and decision change was 1.5 out of 2. The evaluation, however, is limited to the Jimma Health Center Maternal and Childcare Unit. As a result, in addition to the self-reported computer aided CDSS evaluation, a larger scale evaluation and longitudinal measurements are required, including computer aided CDSS usage frequency, duration, and so on.

## Supplementary Information

**Additional file 1.**

## Data Availability

Although most of the data is already available in the manuscript, the identified participant-level data will be made available upon request in return for a signed data access agreement. Please email to the corresponding author for access.

## Footnotes

1 https://github.com/gel1has3/AI_Enabled_CDSS_Evaluation_Framework
