# Supplementary material for: Evaluating a clinical decision support point of care instrument in low resource setting
Source: BMC Med Inform Decis Mak. 2023 Mar 30;23:51. doi: 10.1186/s12911-023-02144-0 (PMC10064719; doi:10.1186/s12911-023-02144-0)
Supplement: Supplementary file 1 — Additional file 1. [file 12911_2023_2144_MOESM1_ESM.pdf]

Evaluating clinical decision support point of care instrument in low resource setting

1. OBJECTIVE

The goal of this study is to evaluate the user acceptance of a clinical decision support (CDS) instrument at the point of care (POC) in low-resource settings (LRS).

2. METHODS

2.1. SITE SELECTION

The evaluation will be carried out at Jimma Health Center. During the need analysis, we studied the clinical guidelines, patient card-sheet, and referral-out registration logbook at Jimma health center. The health center acts as a focal point by handling both inpatient and outpatient cases. It accepts referral cases from community health posts as well as refers referral cases and assigns patients to the primary hospital (Jimma University Specialized Hospital and Shanan Gibe General Hospital).

2.2. STUDY PARTICIPANTS AND PROCEDURES

Caregivers who work at Jimma Health Center in the maternal and child healthcare unit or department will be eligible to participate in the evaluation experiment. The evaluation procedure for the CDS instrument will be carried out after the clinical decision is made rather than during the real-time decision-making process. Therefore, the use of the CDS instrument will have no impact on the care provided.

2.3. EVALUATION DESIGN

The CDS system was developed and deployed on a Raspberry Pi 4 Model B, which has a quad-core 64-bit processor and 4GB of RAM. The CDS instrument WEB-APP is accessed via a smart phone's mobile data (or wireless network). The purpose of the system was to promote high-quality care and assist healthcare workers in identifying referral and locally treatable cases, as well as patient care activities.

The framework for evaluating an artificial intelligence–enabled clinical decision support system was developed based on Ji, Mengting, et al.2021[1] and has been customized to our needs. We adopted 22 of the 28 parameters from the evaluation framework. Outcome changes, service quality, and productivity (related to process change) will not be considered because the CDS instrument will be evaluated after the clinical decision is made. Furthermore, since the variables "information satisfaction, service, and system's quality" were difficult to distinguish, we aggregated them as overall quality.

To set up an experiment for evaluation, the following activities will be carried out:

The screening for participant eligibility and contacting the person of interest, as well as setting up meeting specifics such as time and location, will take place. To provide for as much participant flexibility as feasible, evaluation will be done on participant spare-time, because the number of health professionals at the health center's maternal and child healthcare unit is limited (approximately five to eight) and they are too busy to complete their ordinary daily activities. As a result, instead of an instant patient-by-patient evaluation, the evaluation will be completed over the course of a half-day.

After clarifying the goal and obtaining consent, the participants will be given a guide with detailed step-by-step instructions how to use WEB-APP to assist them in better preparing for the activity.

---

[1] *Ji, M., Genchev, G. Z., Huang, H., Xu, T., Lu, H., & Yu, G. (2021). Evaluation Framework for Successful Artificial Intelligence–Enabled Clinical Decision Support Systems: Mixed Methods Study. Journal of medical Internet research, 23(6), e25929.*

The think-aloud protocol will be followed while the participant attempts to use WEB-APP. The system will be evaluated after the clinical decision was made using the concurrent think-aloud approach. In a thinking aloud test (TA-Test), participants are asked to use the CDS instrument while continuously thinking out loud[1].

In the end, the participant will complete the questionnaire (*found on pages 3 and 4 of this document*). It is a form of psychometric response scale[2] in which respondents express their level of agreement to a statement in five scores: (1) strongly disagree; (2) disagree; (3) neutral; (4) agree; and (5) strongly agree. The questionnaire is structured into five sections with a total of 22 questions to validate and measure the instrument's characteristics in the following order: ease of use (6/22), system quality (2/22), information quality (2/22), decision changes (2/22), process changes (5/22), and user acceptance (5/22).

The questionnaire was translated into Amharic. A freelance and experienced translator then reviewed the translated questionnaire to resolve any discrepancies between the original English version and the translated Amharic questionnaire. The questionnaire will be accessible for submission through mobile, laptop, or paper-based format. We prefer mobile or laptop-based formats to paper-based formats, unless in exceptional instances.

In conclusion, the findings will be analyzed to gain insights and uncover common patterns in order to identify future actions. Moreover, personal information exclusively used for questionnaire verification did not appear in reporting or results. In general, we are committed to protecting your personal information and respecting your privacy.

3. GENERAL INSTRUCTIONS FOR FILLING THE QUESTIONNAIRE

The information you provide will be used solely to test and evaluate the WEB-APP CDS instrument. The data will be valuable in improving the instrument.

The following are statements for evaluating CDS POC in LRS on which some people agree and others disagree. We would like to indicate your opinion after each statement by putting an "**X**" in the box that best indicates the extent to which you strongly disagree, disagree, neutral, agree, or strongly disagree. Furthermore, when the participant's response is “strongly disagree, disagree, or neutral”, additional explanations will be requested, which will be reviewed later for further WEB-APP improvement. The information may be found in the column labeled "*comment in the case of neutral, disagree, and strongly disagree*”.

---

[2] *5-Point Likert Scale. In: Preedy V.R., Watson R.R. (2010) (eds) Handbook of Disease Burdens and Quality of Life Measures. Springer, New York, NY.* https://doi.org/10.1007/978-0-387-78665-0_6363

DATE:________________________________

START TIME: Hour:__________Minute:_________Second:___________

EVALUATOR FULL NAME: ____

			Strongly Disagree	Disagree	Neutral	Agree	Strongly Agree	Comment in the case of neutral, disagree and strongly disagree
Perceived Ease of Use	*1. Learnability*	*I'm satisfied with WEB-APP. I can quickly (i.e. without taking time) learn how to use it and begin doing some work with it.*						
	2. Operability	*I'm satisfied with the amount of effort and time required for the WEB-APP to operate and perform tasks correctly.*						
	3. User Interface	*The WEB-APP allows users to easily navigate through the user interfaces (UI) and deliver the results in the correct format. In addition, the WEB-APP provides appropriate controls, menus, and outputs.*						
	4. Data Entry	*The WEB-APP provides a simple and consistent data entry interface. The WEB-APP enables the user to choose a specific task and encourages minimum data entry and steps through the use of selection boxes, wizards, and other features.*						
	5. Advice to display	*I am satisfied with the advice to display. It refers to the WEB-APP making recommendations to the user, and the users are expected to make their own decisions and overruling WEB-APP recommendations they believe to be inappropriate.*						
	6. Legibility	*I am satisfied how WEB-APP and its decisions are understood by non-AI experts.*						
System Quality	*7. Response time*	*I am satisfied with the response time i.e. it refers to the time taken to transmit the inquiry, process it by WEB-APP, and transmit the response back to the user.*						
	8. Stability	*I am satisfied with the WEB-APP sessions that are crash-free, i.e. as effectively the system handles exceptions and errors.*						
Information Quality	*9. Security*	*I am satisfied with the capability of WEB-APP to protect information and data so that unauthorized persons or systems cannot read or modify them and authorized persons or systems are not denied access.*						
	10. CP Performance	*The WEB-APP enables me to execute activities with appropriate evidence and within acceptable time frames. The WEB-APP makes it easier and more convenient to gather evidence than paper-based clinical guidelines, point-of-care instruments, card-sheet, and so on.*						
Decision Changes	*11. Change in order behavior*	*I am satisfied with the the capability of WEB-APP allowing for real time-based interactions between the user and the WEB-APP recommendations.*						

			Strongly Disagree	Disagree	Neutral	Agree	Strongly Agree	Comment in the case of neutral, disagree and strongly disagree
	12. Change in CP	*The evidence that I got from the WEB-APP is diverse enough and important. The result of using the system are apparent to me.*						
Process Changes	13. Effectiveness	*I am satisfied with the way WEB-APP speeds up workflow and displays the desired output as the user expecst, so that a user can complete tasks accurately and completely in a specified context?*						
	14. Overall usefulness	*Overall, I found the WEB-APP tool was useful. i.e. it refers to the quality of WEB-APPbeing useful under different contextual factors.*						
	15. Adherence to standards	*The evidence that I got from WEB-APPadheres to standards i.e. quality of WEB-APP abiding/ sticking by both industry regulations and government legislations*						
	16. Medical quality	*The quality of evidence that I got from the WEB-APP instrument is high and important*						
	17. User knowledge and skills	*WEB-APP assists or increases the likelihood of desired clinical outcomes and is consistent with current professional knowledge and skills.*						
Acceptance	18. Usage	*My experience with WEB-APP didn't face any problem*						
	19. Expectations confirmation	*I found WEB-APP performed as expected based on my prior experience and/or expectations.*						
	20. Satisfaction of over quality	I am satisfied with the overall WEB-APP's system, service (in finding referral and treatable cases), and information output quality.						
	21. Overall satisfaction	*Overall I'm satisfied with WEB-APP i.e. the affective reactions of users toward the use of WEB-APP in general.*						
	22. Intension to use	*I intend to use the system for my routine duty to perform my job. Given that I have access to WEB-APP for my routine duty, I predict that I would adopt it.*						

END TIME: Hour:__________Minute:_________Second:___________
